# Feedback-Based Treatments for Eating Disorders and Related Symptoms: A Systematic Review of the Literature

**DOI:** 10.3390/nu10111806

**Published:** 2018-11-20

**Authors:** Claudio Imperatori, Miranda Mancini, Giacomo Della Marca, Enrico Maria Valenti, Benedetto Farina

**Affiliations:** 1 Department of Human Sciences, European University of Rome, Via degli Aldobrandeschi 190, 00163 Roma, Italy; mancini.miranda@gmail.com (M.M.); enricomaria.valenti@gmail.com (E.M.V.); benfarina@gmail.com (B.F.); 2 Sleep Disorders Unit, Institute of Neurology, Catholic University, 00153 Rome, Italy; giacomodellamarca@gmail.com; 3 Department of Dynamic and Clinical Psychology, Sapienza University of Rome, 00185 Rome, Italy

**Keywords:** biofeedback, EEG-Neurofeedback, fMRI-Neurofeedback, eating disorders, psychophysiology, eating disorders-related symptoms

## Abstract

The effectiveness of biofeedback and neurofeedback has been investigated in a range of psychiatric disorders. However, to date, there are few studies on the clinical usefulness of feedback-based techniques for eating disorders (EDs) and EDs-related symptoms (e.g., food craving). A systematic search of PubMed, Scopus and PsychINFO identified 162 articles. Among these, thirteen studies exploring the therapeutic use of biofeedback and neurofeedback in EDs or EDs-related symptoms were included. Biofeedback and neurofeedback were implemented respectively in five and eight of all reviewed articles. No studies incorporated different feedback modalities or both biofeedback and neurofeedback. The considered studies provide preliminary data of the usefulness of feedback-based techniques in the treatment of several dysfunctional eating behaviors (e.g., food craving, rumination). Although no significant effect has been reported for other important EDs-related symptoms (i.e., body image disturbance), feedback-based techniques are also associated with significant modifications of both sympathetic reaction to food-related stimuli and brain activity in several regions of the reward system (e.g., insula). Taken together the results of the present review suggest that feedback-based treatments may be useful in the treatment of several dysfunctional eating behaviors operating both on top-down and bottom-up individual coping strategies. Methodological and clinical issues are also discussed.

## 1. Introduction 

Eating disorders (EDs) are severe and disabling conditions caused by multiple factors (e.g., genetic and psychosocial) [1], which are associated with significant functional impairments [2], high mortality risk [3] and treatment difficulties [4]. The last edition of the Diagnostic and Statistical Manual of Mental Disorders (DSM-5) [5] includes a substantially revised section on EDs [6,7], now named “Feeding and Eating Disorders”. This section includes the following diagnoses: pica, rumination disorder (RD), avoidant/restrictive food intake disorder (ARFID), anorexia nervosa (AN), bulimia nervosa (BN), binge eating disorder (BED), and other specified feeding or eating disorder (OSFED). 

EDs and related symptoms are common in both children and adolescents [8,9]. In adults, the lifetime prevalence is about 0.6% for AN, 1% for BN, and 3% for BED [2,3]. Furthermore, it has been recently reported that, following the transition to DSM-5, the prevalence of OSFED has decreased, and that AN, BN and BED have increased in non-clinical samples [10]. Similarly, EDs-related symptoms, such as food craving (i.e., an intense desire to consume a specific food that is difficult to resist); Refs. [11,12,13] or loss of control over eating, are widely reported amongst the general population. For example, in a non-clinical sample it has been reported [14] that 28% of women and 13% of men reported food craving at least once a week during the past 6 months. This symptom is also commonly experienced in AN [15,16], BN [16,17,18,19], and BED [20,21]. Moreover, it has been reported that food craving severity is positively associated with both body mass index (BMI) [13,22,23,24,25], drop-out from weight loss programs [25,26,27], and a meta-analysis [28] on 3292 individuals showed that it significantly contributes to dysfunctional eating behaviours and weight gain.

Similarly, loss of control over eating is a relatively frequent experience among both adolescents from the general population (i.e., about 17%) [29], adults with obesity (i.e., BMI ≥ 30 kg/m^2^) and patients with EDs [30]. Additionally, it was positively associated with other psychopathological symptoms (e.g., non-suicidal self-injury, body dissatisfaction, higher depressive symptoms) [31].

Multidisciplinary approaches, combining medical, dietetic and psychological interventions are generally recommended in the treatment of EDs [32]. However, it is known that current treatments for EDs are often related to treatment difficulties [4] and poor long-term efficacy [1,33,34]. For example, it has been reported that several treatments, such as nutritional rehabilitation and psychological approaches (e.g., cognitive-behavioral therapy, family-based therapy), are associated with high relapse rates in adolescents with AN [33]. Similarly, binge eating remission rates at the end of treatments are only found in 30–40% of patients with BN [1]. Therefore, the necessity of additional treatment modalities for these mental disorders has been proposed [35]. 

Feedback-based treatments (i.e., biofeedback and neurofeedback) have been suggested as additional treatment modalities for EDs [36]. Feedback is considered a crucial component of mental health interventions, increasing motivation, facilitating the learning process and modifying dysfunctional thoughts and behaviors [37]. Over the last thirty years there has been an increasing number of disorders, including EDs, for which biofeedback and neurofeedback have been investigated with more or less empirically supported results [38,39]. 

Biofeedback and neurofeedback are operant-conditioning based trainings that allow individuals to learn how to regulate neurophysiological activity in response to real-time feedback, in order to improve health and performance [38]. Biofeedback refers to an intervention that helps individuals to control or change their physiologic functioning (e.g., heart rate, electrodermal activity, respiratory rate, muscle tension and peripheral temperature) [40]. Neurofeedback refers to a form of biofeedback involving different modalities, such as electroencephalography (EEG) or real-time functional magnetic resonance imaging (rt-fMRI), that trains individuals to control or modify their brain activity [41]. Compared to other neuro-stimulation techniques (i.e., transcranial direct current stimulation), which may be associated with several mild and transient adverse effects [42], feedback-based treatments are not related with side effects [43] and seems to be easy and affordable techniques for general practices and clinicians [44,45]. 

During a typical feedback-based session, neurophysiological activity (e.g., brain and or electrodermal activity) is “fed back” to the individual using a brain-computer interface, providing continuously updated information about their success in regulating their neurophysiological parameters [36]. The successful self-regulation of the individuals’ physiology represents an immediate and effective positive reinforcement, creating a positive loop between the machine’s feedback and the patient’s successful self-regulation [40]. Therefore the three necessary components for a feedback-based session are: (i) a therapist explaining the equipment and its use, (ii) a patient and (iii) a monitoring machine that provides accurate neurophysiological information [38]. Although training sessions and modalities may be different according to the individual needs and/or the diagnosis [40], general protocol guidelines are provided [38].

## 2. Study Rationale

The clinical efficacy of biofeedback and neurofeedback has been investigated in a range of psychiatric disorders [38,39]. However, to date, there are few studies on the clinical usefulness of feedback-based techniques for EDs or EDs-related symptoms. Therefore, the present systematic review (PRISMA checklist is reported in Table S1) was carried out to explore the current therapeutic use of biofeedback and neurofeedback in EDs and EDs-related symptoms (i.e., food craving, binge eating, loss of control over eating). Specifically, in this study we investigated: (i) the type of feedback-based protocol applied, and (ii) the usefulness of feedback-based techniques assessed through clinical scales and/or neurophysiological measures.

## 3. Methods

### 3.1. Inclusion/Exclusion Criteria 

We used the P.I.C.O.S. (Population, Interventions, Comparators, Outcomes, and Study Design) criteria to identify relevant studies. This review focused on feedback-based interventions for EDs and related symptoms. Therefore, original articles reporting data from studies investigating the potential therapeutic effects of different biofeedback (i.e., heart rate, electrodermal activity, respiratory rate, muscle tension and peripheral temperature) and neurofeedback modalities (i.e., EEG, fMRI) in this field were initially considered. Studies involving children and adolescents were also taken into consideration. Book chapters, conference papers, reviews, dissertations, and case reports were not included. Articles in which either feedback-based treatments for EDs or EDs-related symptoms were not the focal point were also excluded. Moreover, feedback-based studies investigating EDs-related symptoms (i.e., food craving, binge eating, overeating) in non-clinical or subthreshold sample were initially considered. Both randomized and non-randomized controlled studies, as well as pre-/post-intervention comparison reports were considered.

According to guidelines for the evaluation of the clinical efficacy of psychophysiological interventions [46], as well as with previous systematic reviews focused of feedback-based treatments in psychiatric disorders [39,47], articles that provided the following information were included in the study: (i) feedback modality type, (ii) sample (i.e., including age, sex, medication status, BMI, recruitment), (iii) study design (including type, number and duration of protocol sessions, as well as description of study conditions), (iv) collection and analysis of neurophysiological (e.g., EEG power spectrum, electrodermal activity etc.), behavioral (e.g., number of binge episodes) and/or psychological outcomes measure (i.e., clinical scales). Furthermore, according to Schoenberg and David [39], studies that did not report 2 or more components of points (ii) and (iii), and/or articles that did not specify points (i), (iv) and (v) were excluded.

### 3.2. Search Strategy

**Figure 1:**
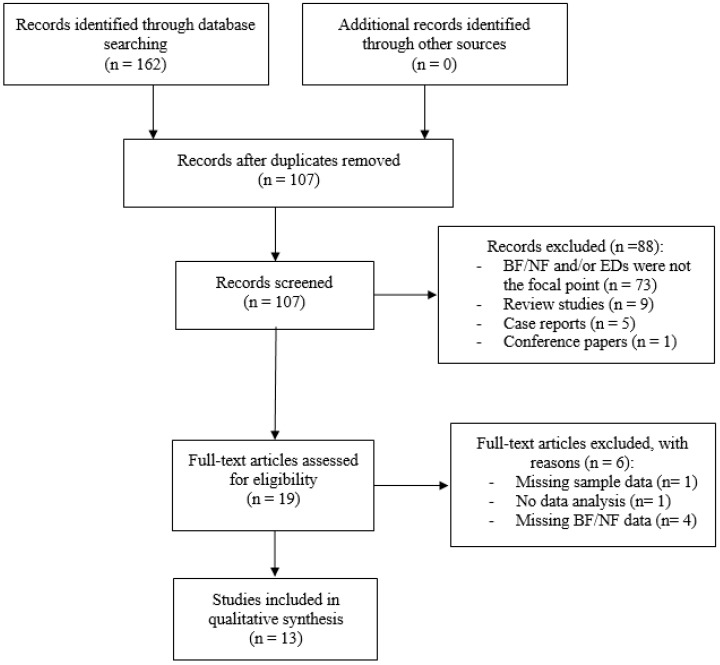
Flow diagram of selection of studies. Abbreviation: BF = Biofeedback; NF = Neurofeedback; EDs = Eating Disorders.

Potentially relevant studies were initially identified by searching publications from the year 1970 to 5 August 2018 through the following databases: PubMed, Scopus and PsychInfo. Only English articles were taken into account. The following search terms were entered into the databases: “biofeedback” OR “neurofeedback” AND “anorexia nervosa”, “bulimia nervosa”, “binge eating disorder”, “pica”, “rumination disorder”, “avoidant/restrictive food intake disorder”, “food intake disorder”, “food craving”, “binge eating”, “overeating”, “eating psychopathology”. Articles resulting from the search strategy were examined for relevance by screening titles and abstracts. Then, articles that appeared to meet inclusion criteria were further evaluated by two independent researchers (C.I. and M.M.) in order to assess all inclusion/exclusion criteria. In case of disagreement, a senior researcher (B.F.) resolved any discrepancies and decided whether or not the study was going to be included. A detailed flow diagram of selection of studies is reported in Figure 1.

## 4. Results 

The initial search resulted in 162 articles. Duplicate articles were eliminated, leaving 107 screened studies. Of these articles, 19 met initial inclusion criteria and were assessed for eligibility. Six articles were subsequently excluded with reasons (e.g., missing information pertaining to methods/outcome measures, no description of biofeedback modality, no statistical analysis etc.). 

Thirteen articles fulfilled the quality assessment and are considered in this review (Table 1). 

Biofeedback and neurofeedback were implemented respectively in five and eight of all reviewed articles. No studies incorporated different feedback modalities (e.g., electrodermal and electromyography biofeedback) or both biofeedback and neurofeedback. None of the studies investigated in this review reported relevant side effects for both biofeedback and neurofeedback training. 

Mean number of sessions per study was 7.42 (range 1–12). Among reviewed article, three studies were focused on non-clinical samples [55,56,57], two on subthreshold samples [58,59], two on adolescents with AN [60,61], two on patients with RD [62,63] and 5 on overweight/obese individuals [61,64,65,66,67].

Eight studies were randomized [56,57,58,59,60,64,65,67], but only one was a double-blind trial [65]. Four studies did not have a control condition [55,61,63,66]. No treatment/waiting list was the most frequently (*n* = 7) control condition used [56,57,58,59,62,64,67], and only one study implemented a placebo condition (i.e., simulation protocol) [65] or an alternative treatment [59] (i.e., mental imagery).

Due to the high heterogeneity of samples, outcome measures and feedback modality, a meta-analysis was not performed.

### 4.1. Biofeedback Studies

Biofeedback was investigated in five of all reviewed articles. Of these, two studies implemented electrodermal biofeedback (ED-BF) [61,67], one heart rate variability biofeedback (HRV-BF) [57], one electromyography biofeedback (EMG-BF) [62], and one diaphragmatic breathing biofeedback (DB-BF) [63]. 

Barba et al. [62] and Halland et al., [63], investigated the usefulness of EMG-BF and DB-BF in patients with RD respectively. Both studies reported significant changes in clinical (e.g., decrease in regurgitation and rumination episodes) and physiological (e.g., decrease of intragastric pressure) outcomes.

Two studies examined the potential therapeutic effects of ED-BF in obese patients with overeating. Pop-Jordanova [61], in a pre-post study design, showed that 5 sessions of ED-BF were associated with a decrease of electrodermal activity in a sample of girls with obesity (*n* = 76) and AN (*n* = 27). In a sample of obese women (*n* = 30), Teufel et al. [67], through a randomized controlled study, reported that compared to a waiting list, both ED-BF focused on food stimuli and ED-BF focused on unspecific stimuli were associated with an increased ability to tolerate food-related stress (e.g., decrease of electrodermal activity and increase self-efficacy in dealing with food). Although these results remained stable after 6-months follow-up, no significant changes were observed in BMI and in ED-related symptoms (e.g., tendency to lose control of food intake).

Finally, in a study on non-clinical sample of cravers (*n* = 56), Meule et al., [57], reported that, compared to a waiting-list, 12 sessions of HRV-BF were associated with a decrease of food craving as well as with a decrease of eating and weight concerns. No significant changes were observed in physiological measures (e.g., HRV) and in other EDs-related symptoms (e.g., shape concern, binge frequency).

### 4.2. Neurofeedback Studies

Neurofeedback was investigated in eight of all reviewed articles. Of these, six studies implemented EEG [56,58,59,60,64,65] and two rt-fMRI [55,66].

Two randomized controlled studies investigated the usefulness of EEG beta training neurofeedback (i.e., decrease beta activity at Cz electrode) in female subthreshold samples of restrained eaters (*n* = 27) [58] and binge eaters (*n* = 57) [59]. Both studies reported that, compared to a waiting-list, 10 sessions of neurofeedback were associated with a decrease in overeating episodes and related distress. These results remained stable after 3-months follow-up.

Two randomized controlled studies investigated the effectiveness of EEG alpha/theta training neurofeedback (i.e., raise posterior theta over alpha amplitude with eyes closed without falling asleep) in reducing food craving, in a non-clinical sample (*n* = 50) [56] and in a sample of overweight women (*n* = 30) respectively [64]. It has been observed that, compared to a waiting-list, 10 sessions of neurofeedback were associated with a decrease of food craving severity [56,64] as well as with an improvement of mental health [64]. A significant increase of resting EEG alpha power in several brain areas involved in food craving (e.g., insula) and food cue reactivity (e.g., parahippocampal gyrus) was also documented [56]. Although changes in food craving persisted after 4-months follow-up, no significant modifications were reported in other EDs-related symptoms (e.g., weight and shape concerns) and in the general level of psychopathology [56].

Lackner et al. [60] examined the potential therapeutic effects of EEG alpha training neurofeedback (i.e., raise posterior alpha activity) in female adolescents with AN (*n* = 22). It has been observed that, compared to the standard treatment, 10 sessions of neurofeedback were associated with an improvement in several ED-related symptoms (e.g., decrease of restriction and dieting behavior) and with an increase of emotional competence. Although modifications of resting EEG power were also documented (i.e., increase of theta power), no significant modifications were reported in psychopathological symptoms (with the exception of interpersonal sensitivity), in BMI and in body image related symptoms.

Two studies investigated the usefulness of rt-fMRI neurofeedback during the exposure to appetitive food pictures, respectively in a non-clinical sample of women (*n* = 10) [55] and in overweight/obese men (*n* = 8) [66]. Both studies showed that rt-fMRI neurofeedback could modify brain activity [55] and brain connectivity [66] in crucial areas involved in the reward system (e.g., amygdala and prefrontal cortex). Although no significant improvement was reported in food craving or in calorie intake assessment, a decrease of subjective hunger [55] and negative mood [66] related to food stimuli was reported. 

Finally, in the only double-blind, placebo-controlled neurofeedback study, Leong et al. [65], investigated the effectiveness of infraslow EEG neurofeedback (i.e., the modulation of slow wave activity (0–0.1 Hz)) in obese women with food addiction symptoms. Compared to placebo condition, it has been reported that 6 sessions of neurofeedback were associated with an increase of infraslow activity in the posterior cingulate cortex as well as with a decrease of state food craving.

### 4.3. Risk of Bias

**Figure 2:**
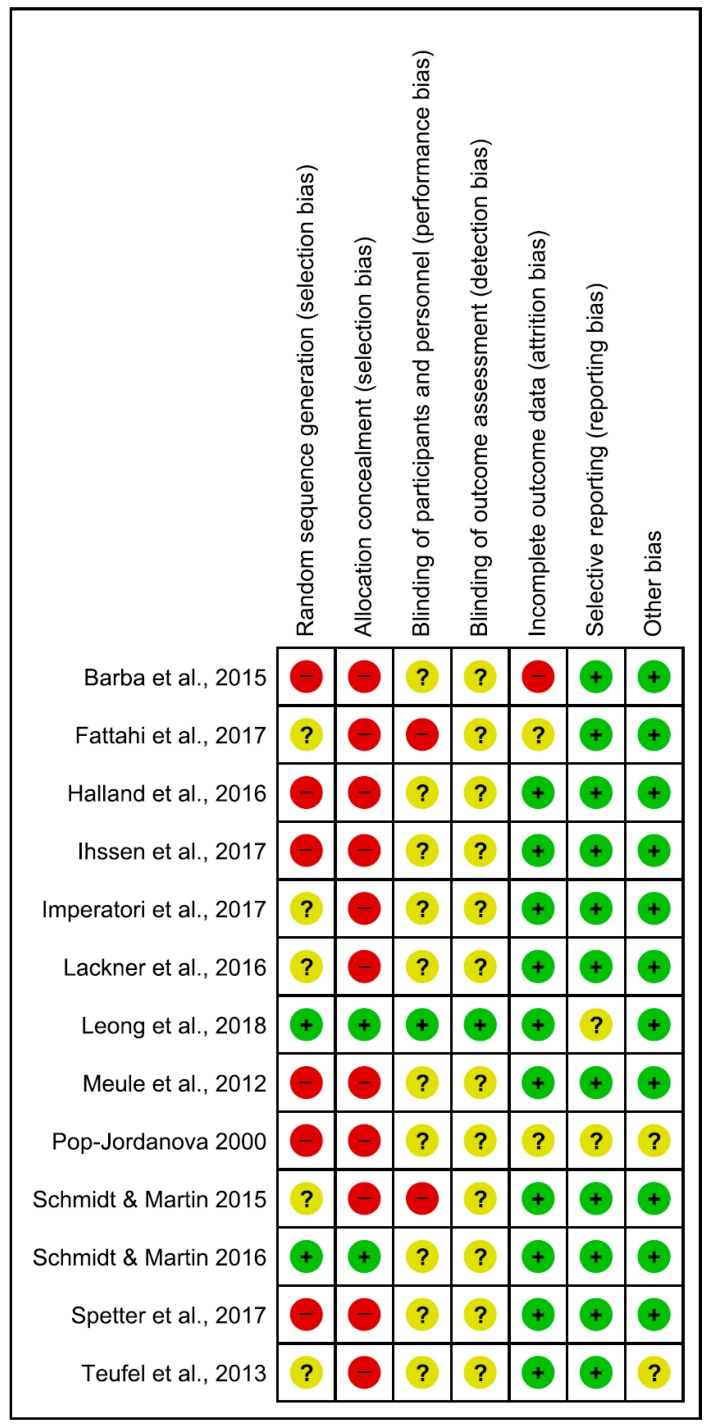
Risk of bias summary: review authors’ judgements about each risk of bias item for each included study. Green, yellow and red circles represent low, unclear and high risk of bias respectively.

**Figure 3:**
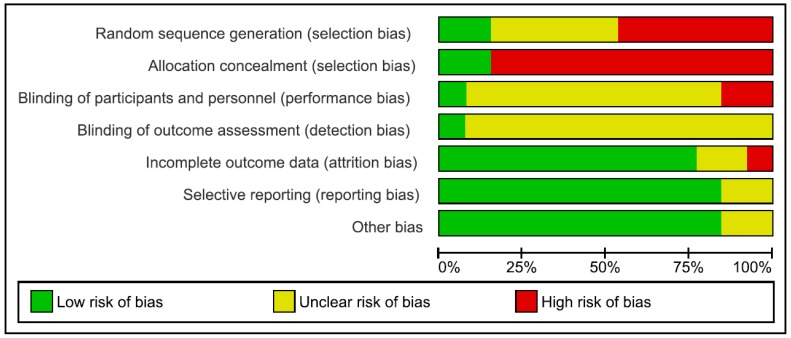
Risk of bias graph: review authors’ judgements about each risk of bias item presented as percentages across all included studies.

Risk of bias in the reviewed articles was performed according to Cochrane standards of practice [68]. Two reviewers (CI and GDM) independently assessed the risk for bias. In case of disagreement, a senior researcher (BF) resolved any discrepancies. Risk for bias was mainly related to the lack of both blinding and control conditions. Assessment of bias in the included studies are reported in Figure 2 and Figure 3.

## 5. Discussion

The main aim of the present systematic review was to report how feedback-based treatment (i.e., biofeedback and neurofeedback) have been used in the treatment of EDs and EDs-related symptoms. To the best of our knowledge, this is the first systematic review that investigates both biofeedback and neurofeedback applications in this field. 

The review identified 13 articles, five focused on biofeedback and eight focused on neurofeedback, providing preliminary data of the usefulness of feedback-based techniques in the treatment of several dysfunctional eating behaviors (Table 2). Specifically, it has been reported that both neurofeedback and biofeedback training may decrease food craving severity [56,57,58,64,65], overeating episodes [58,59], regurgitation [62] and rumination [63] episodes, restricting behavior [60], eating and weight concerns [57]. Furthermore, this review showed that feedback-based techniques are associated with significant modifications of both sympathetic reaction to food-related stimuli [61,62,63,67] and brain activity in several regions of the reward system (e.g., prefrontal cortex, amygdala, insula) [55,56,60,65,66].

These results seem to suggest that both biofeedback and neurofeedback increased the ability to better tolerate stress and the ability to cope with situations involving food. Indeed, the most common goal of feedback-based treatments implemented for EDs and EDs-related symptoms is the reduction of stressful arousal and the increase of top-down control abilities. For example, the aim of EEG alpha training [60] is to enhance individual alpha frequency, which is usually associated with alert relaxation [69], in the parietal area (i.e., Pz electrode). Similarly, the goal of EEG beta training [58,59] is to down-regulate beta activity, which is positively associated with ruminative states of stressful arousal [70]. Consistently, the aim of alpha/theta training is to raise posterior (i.e., Pz electrode) theta over alpha amplitude in order to produce a state of deep relaxation, enhancing top-down mental functions [71], such as mentalization [72]. Finally, an rt-fMRI neurofeedback has been implemented to increase functional connectivity between brain areas (i.e., dorsolateral prefrontal cortex and ventromedial prefrontal cortex) that regulate the top-down control of appetite for high-calorie foods [66]. 

Interestingly, it has also been suggested that the target of neurofeedback is to increase bottom-up strategies in order to decrease the salience attached to food [65], or down-regulate brain regions activation (i.e., amygdala) during exposure to food cues [55]. Therefore, taken together these data suggest that feedback-based treatments implemented for EDs and EDs-related symptoms can operate both on top-down and bottom-up strategies in order to foster the neural mechanisms underlying successful coping during stressful food-related situations.

On the other hand, this review showed no significant effect of feedback-based techniques in improving other important EDs-related symptoms, such as body image disturbance, a crucial core of AN and BN [5], which seems to affect also BED [73]. No significant modifications associated with both biofeedback and neurofeedback were also reported for BMI in both patients with AN and overweigh/obesity. These results confirm the need of multidisciplinary approaches, combining medical, dietetic and different kind of psychological interventions in the treatment of EDs and ED-related symptoms [32]. For example, combining EEG alpha training neurofeedback [60] with cognitive-behavioral exposure-based body image therapy [74] may be useful in the treatment of EDs.

It is also interesting to note that, compared to feedback-based interventions in other psychiatric disorders [39], no studies considered in the present review incorporated different biofeedback modalities or both biofeedback and neurofeedback. Therefore, it is possible that combining HRV-biofeedback [57] and EEG alpha/theta neurofeedback [56,64] may be more effective in reducing food craving severity in both clinical and non-clinical samples. Similarly, the combination of two neurofeedback training methods, such as EEG alpha/theta training and EEG beta training, or the combination of feedback-based techniques with other cognitive-behavioral techniques (e.g., cognitive restructuring, exposure therapy and response prevention) may be more effective in improving a wider range of ED-related symptoms (i.e., binge eating episodes, food craving). 

Although the present review provided preliminary results of the usefulness of feedback-based techniques in the treatment of EDs and EDs-related symptoms, several methodological issue should be considered. 

First, up to date, there are few randomized, double-blind, placebo-controlled studies investigating the effectiveness of both biofeedback and neurofeedback in large clinical samples. Indeed, among reviewed randomized trials (*n* = 8) the most frequent control condition implemented was the no treatment/waiting list, and only one study was double-blind. Consequently, most of the studies included in the present review had a high risk of bias, which was mostly related to the lack of both blinding and control conditions (e.g., mock feedback). This is in line with a recent systematic review investigating the potential therapeutic effects of neurofeedback training in psychiatric disorders associated with criminal offending [47]. Therefore, future studies should compare biofeedback and neurofeedback with sham procedures in order to rule out the placebo effect. Secondly, compared to feedback-based interventions in other psychiatric disorders [39], the mean number of sessions was relatively lower (i.e., 7.42). Although it has been reported that patients may benefit by undergoing 8 to 12 sessions [40], the possibility that a greater number of sessions (i.e., from 20 to 30) may maximize both biofeedback and neurofeedback results should be assessed. Furthermore, the stability of psychological/behavioral and neurophysiological (e.g., EEG power) outcomes should also be investigated taking into account long-term follow-up (i.e., at least one year). Finally, although the present review did not provide relevant differences associated with both age and gender, future studies should also investigate the relationship between feedback-based interventions outcomes (e.g., adherence to treatment, symptoms improvement) and several socio-demographic data (e.g. adolescent vs adults with EDs, and/or men vs women with EDs). 

### Study Limitations and Conclusions

There are several limitations of the present review that should be considered. Firstly, the search strategy was limited to articles published in English. Secondly, due to the high heterogeneity of samples, outcome measures and feedback modalities, a meta-analysis in order to quantify the effectiveness of both biofeedback and neurofeedback was not performed. 

Despite these limitations, to the best of our knowledge, this is the first systematic review that investigates both biofeedback and neurofeedback applications in the treatment of EDs and EDs-related symptoms. In conclusion, the results of the present review suggest that, although future studies are needed in order to draw definitive conclusions, feedback-based techniques may be useful in the treatment of several dysfunctional eating behaviors (e.g., food craving, binge eating) operating both on top-down and bottom-up individual coping strategies. 

## Figures and Tables

**Table 1:** Characteristics of the included studies (*n* = 13).

Authors	Participants	Design		Outcome Measures	Main Results
	(a) Sample (b) N (sex) (c) Age (mean ± sd. or range) (d) BMI (mean ± sd. or range) (e) Psychotropic medication (yes/no)	(a) Group (type of intervention) (b) Randomized (yes/no) (c) Blind (single/double)	(d) N of sessions (e) Duration (f) Follow-up (yes/no)	(a) Psychological/Behavioral (b) Physiological	(a) Psychological: ↑ = sign. increase; ↓ = sign. decrease; x = no change (b) Physiological: ↑ = sign. increase; ↓ = sign. decrease; x = no change (c) Stability at follow-up (yes/no)
**Biofeedback Studies**					
Pop-Jordanova 2000	(a) preadolescents with obesity; preadolescents with AN (b) 76 (F); 27 (F) (c) 12.75 ± 1.75; 14.25 ± 2.99 (d) bodyweight over 95th percentile; bodyweight below the 3rd percentile (e) n/a	(a) one group: ED-BF and supportive psychotherapy (b) no (c) n/a	(d) 5 (e) n/a (f) no	(a) n/a (b) EDA	(a) n/a (b)↓ of EDA in children with obesity and AN (c) n/a
Meule et al., 2012	(a) non-clinical sample (b) 56 (47 F, 9 M) (c) 24.12 ± 3.79 (d) 22.65 ± 3.19 (e) n/a	(a) 3 groups: (i) BFG (*n* = 14 high cravers): HRV-BF (ii) CG_1_ (*n* = 14 high cravers): no intervention (iii) CG_2_ (*n* = 28 low cravers): no intervention (b) pseudo-randomization * (c) single	(d) 12 (e) 20 min. (f) no	(a) FC ^1^; eating disorder symptoms ^2^; FA ^3^; self-regulatory success in dieting; emotion regulation; locus of control (b) HR; HRV	(a) ↓ FC only in BFG; ↓ in eating and weight concerns only in BFG; x in other eating disorder symptoms, emotion regulation, FA, locus of control and self-regulatory success in dieting (b) ↓ in HRV in CG_1_ (c) n/a
Teufel et al., 2013	(a) obese individuals (b) 30 (F) (c) 48.67 ± 11.93 (d) 35.5 ± 5.3 ** (e) no	(a) 3 groups: (i) BFG_1_ (*n* = 10): ED-BF focused on food stimuli (ii) BFG_2_ (*n* = 10): ED-BF focused on unspecific food stimuli (iii) CG (*n* = 10): no intervention (b) yes (c) single	(d) 8 (e) 21 min. (f) 3 months	(a) self-efficacy, stress, ability to relax, eating disorder symptoms^4^; BMI (b) EDA to food stimuli	(a) ↑ of food-related self-efficacy and perceived stress in BFGs; ↑ ability to relax in BFGs; x in BMI and, eating disorder symptoms (b) ↓ in EDA to food stimuli in BFGs (c) yes (a + b); ↓ loss of control during eating in BFG_2_
Barba et al., 2015	(a) patients with RD (b) 28 (17 F, 11 M) (c) age range (14–76) (d) 22 ± 2 (e) yes (*n* = 4)	(a) 2 groups: (i) BFG (*n* = 15): EMG-BF (ii) CG (*n* = 4): no intervention (b) n/a (c) single	(d) 3 plus instructions for daily exercise (e) n/a (f) 1, 3, 6 months	(a) regurgitation episodes and associated abdominal symptoms. (b) EMG of abdominothoracic muscles	(a) ↓ in regurgitation episodes and associated abdominal symptoms immediately after treatment and after 6 month follow up (b) ↓ of intercostal and anterior wall muscle activity (c) yes (a + b)
Halland et al., 2016	(a) patients with RD (b) 16 (9 F, 7 M) (c) 37 ± 13 (d) 26.5 ± 5 (e) yes	(a) one group: DB-BF (b) no (c) n/a	(d) n/a (e) n/a (f) no	(a) rumination episodes (b) postprandial intragastric and esophagogastric junction pressure	(a) ↓ rumination episodes (b) ↑ of esophagogastric junction pressure; ↓postprandial intragastric pressure (c) n/a
**Neurofeedback Studies**					
Schmidt & Martin 2015	(a) subthreshold sample of restrained eaters (b) 27 (F) (c) 34.54 ± 10.40 ** (d) 27.35 ± 5.24 ** (e) no	(a) 2 groups: (i) NFG (*n* = 14): EEG beta training NF (↓ 23–28 Hz activity at Cz) (ii) CG (*n* = 13): no intervention (b) yes (c) single	(d) 10 (e) 45 min. (f) 3 months	(a) overeating episodes and related distress; FC ^1^; perceived dieting success; perceived stress; well-being (b) n/a	(a) ↓ overeating episodes and related distress in NFG; ↑ of perceived dieting success in NFG; x in FC, perceived stress, and well-being (b) n/a (c) yes (overeating episodes and related distress); ↓ of FC in NFG
Lackner et al., 2016	(a) adolescents with AN (b) 22 (F) (c) age range (12–18) (d) 15.41 ± 1.66 ** (e) yes (*n* = 1)	(a) 2 groups: (i) NFG (*n* = 10): EEG alpha training NF (↑ 8–12 Hz activity at Pz) and usual maintenance treatment CG (*n* = 12): usual maintenance treatment (b) yes (c) single	(d) 10 (e) 20 min (f) no	(a) psychological wellbeing, eating disorder symptoms ^4,5,6^, emotional competence, psychopathology (b) resting EEG power	(a) ↑ of disinhibition, ↑ of hunger;↓ restriction and dieting in NFG; ↓ of interpersonal sensitivity and ↑ emotional competence in NFG; x in BMI and psychopathology (b) ↑ EEG theta power (eyes closed) in NFG; x in EEG alpha power (c) n/a
Schmidt & Martin 2016	(a) subthreshold sample of binge eaters (b) 57 (F) (c) 44.77 ± 15.15 (d) 28.77 ± 5.47 (e) no	(a) 3 groups: (i) NFG (*n* = 18): EEG beta training NF (↓ 23–28 Hz activity at Cz) (ii) CG_1_ (*n* = 18): mental imagery (iii) CG_2_ (*n* = 21): no intervention (b) yes (c) single	(d) 10 (e) 45 min (f) 3 months	(a) subjective binge eating episodes and related, perceived stress, FC ^7^, self-efficacy (b) n/a	(a) ↓ binge eating episodes in NFG; ↓ of distress resulting from binge in NFG and CG_1_; ↓ of FC in NFG and CG_1_; ↓ of perceived stress and ↑ of dietary self-efficacy in NFG (b) n/a (c) yes (↓ binge eating episodes and related distress)
Ihssen et al., 2017	(a) non-clinical sample (b) 10 (F) (c) 21.4 ± 2.3 (d) 23.53 ± 2.66 (e) no	(a) one group: rt-fMRI-NF during exposure to appetitive food pictures (b) no (c) n/a	(d) 1 (e) 280 s (f) no	(a) subjective hunger, state FC ^8^, general FC ^9^, feelings of satiety (b) functional activation in brain areas	(a) ↓ of subjective hunger; ↑ of state FC; x in feelings of satiety; x in general FC (b) ↓ activation in reward brain areas (e.g., amygdala, insula, PFC) (c) n/a
Imperatori et al., 2017	(a) non-clinical sample (b) 50 (36 F, 14 M) (c) 22.90 ± 2.68 (d) 21.93 ± 3.41 (e) no	(a) 2 groups: (i) NFG (*n* = 25): EEG alpha/theta training NF (↓ 8–12.5 Hz and ↑ 4.5–7.5 Hz activity at Pz) (ii) CG (*n* = 25): no intervention (b) yes (c) single	(d) 10 (e) 27 min (f) 4 months	(a) FC ^1^, eating disorder symptoms ^2^, psychopathology (b) resting EEG power	(a) ↓ in FC in NFG; x in eating disorder symptoms and psychopathology (b) ↑ of alpha power in reward brain areas (e.g., insula, parahippocampal gyrus) (c) yes (a; b was not assessed)
Fattahi et al., 2017	(a) overweight (b) 30 (F) (c) age range (20–50) (d) n/a (e) n/a	(a) 2 groups: (i) NFG (*n* = 15): EEG alpha/theta training NF (↓ 8–12.5 Hz and ↑ 4.5–7.5 Hz activity at Pz) (ii) CG (*n* = 15): no intervention (b) yes (c) single	(d) 10 (e) 30–45 min (f) no	(a) FC ^1^ and mental health (b) n/a	(a) ↓ in FC and ↑ mental health in NFG (b) n/a (c) n/a
Spetter et al., 2017	(a) overweight/obese individuals (b) 8 (M) (c) 31.8 ± 4.4 (d) 29.4 ± 1.4 (e) no	(a) one group: rt-fMRI-NF during exposure to appetitive food pictures (b) n/a (c) n/a	(d) 4 (e) 9 min (f) no	(a) hunger and mood related ratings, food choice task, calorie intake (b) brain functional connectivity	(a) x in food choice task and in calorie intake assessment; x in hunger, fullness, satiety and appetite; ↓ in fear and agitation (b) ↑ functional connectivity between dlPFC and vmPFC (c) n/a
Leong et al., 2018	(a) obese individuals with FA symptoms (b) 21 (F) (c) 43.01± 13.97 ** (d) 33.5 ± 7.44 ** (e) no	(a) 2 groups: (i) NFG (*n* = 11): infraslow EEG-NF (modulation of slow wave activity at the PCC) (ii) CG (*n* = 10): placebo (b) yes (c) double	(d) 6 (e) 10 min (session #1) 20 min (other sessions) (f) 4 weeks	(a) state FC ^10^ (b) resting EEG power	(a) ↓ of state FC (b) ↑ in infraslow activity in the PCC (c) n/a

Abbreviations: *n* = number; BMI = body mass index; AN = anorexia nervosa; F = females; ED-BF = electrodermal biofeedback; n/a = not applicable; EDA = electrodermal activity; M = males: BFG = biofeedback group; HRV-BF = heart rate variability biofeedback; CG = control group; FC = food craving; FA = food addiction; HR = heart rate; HRV = heart rate variability; RD = rumination disorder; EMG-BF = electromyography biofeedback; EMG = electromyography; DB-BF = diaphragmatic breathing biofeedback; NFG = neurofeedback group; EEG = electroencephalography; rt-fMRI-NF = real-time functional magnetic resonance imaging neurofeedback; PFC = prefrontal cortex; dlPFC = dorsolateral prefrontal cortex; vmPFC = ventromedial prefrontal cortex; PCC = posterior cingulate cortex. Notes: * = 3 participants that could not participate in the study because of time constraints were then assigned to the control group; ** = pooled standard deviation; ^1^ = assessed with the Food Craving Questionnaire-Trait [48]; ^2^ = assessed with the Eating Disorder Examination Questionnaire [49]; ^3^ = assessed with Yale Food Addiction Scale [50]; ^4^ = assessed with Three Factor Eating Questionnaire [51];^5^ = assessed with Eating Disorder Cognition Questionnaire [52]; ^6^ = assessed with Body Image Avoidance Questionnaire [53]; ^7^ = assessed with the Food Craving Questionnaire-Trait-reduced [24]; ^8^ = assessed with a single item (“How strong is your desire to eat?”) rated on a five-point scale; ^9^ = assessed with the modified Trait and State Food Craving Questionnaire [54]; ^10^ = assessed with the Food Craving Questionnaire-State [48].

**Table 2:** Usefulness of feedback-based techniques for the treatment of dysfunctional eating behaviors.

Clinical Eating-Related Problem	Feed-Based Technique	Number of Sessions
Food craving	HRV-BF; Beta-NF; Alpha/Theta-NF; Infraslow-NF	6 to 10
Binge eating episodes	Beta-NF	10
Regurgitation episodes	EMG-BF	3
Rumination episodes	DB-BF	n/a
Restricting behavior	Alpha-NF	10
Eating and weight concerns	HRV-BF; Alpha-NF	12–10

HRV-BF = heart rate variability biofeedback; NF = Neurofeedback; EMG-BF = electromyography biofeedback; DB-BF = diaphragmatic breathing biofeedback; n/a = not applicable.

## Supplementary Materials

Supplementary materials can be found at http://www.mdpi.com/2072-6643/10/11/1806/s1, Table S1: Preferred Reporting Items for Systematic Reviews and Meta-Analyses (PRISMA) checklist.
